# Mecasermin in Insulin Receptor-Related Severe Insulin Resistance Syndromes: Case Report and Review of the Literature

**DOI:** 10.3390/ijms19051268

**Published:** 2018-04-24

**Authors:** Michaela Plamper, Bettina Gohlke, Felix Schreiner, Joachim Woelfle

**Affiliations:** Pediatric Endocrinology and Diabetology Division, Children’s Hospital, University of Bonn, Adenauerallee 119, 53113 Bonn, Germany; Michaela.Plamper@ukbonn.de (M.P.); Bettina.Gohlke@ukbonn.de (B.G.); Felix.Schreiner@ukbonn.de (F.S.)

**Keywords:** severe insulin resistance syndromes, Donohue syndrome, mecasermin, rhIGF-I

## Abstract

Mutations in the insulin receptor (INSR) gene underlie rare severe INSR-related insulin resistance syndromes (SIR), including insulin resistance type A, Rabson–Mendenhall syndrome and Donohue syndrome (DS), with DS representing the most severe form of insulin resistance. Treatment of these cases is challenging, with the majority of DS patients dying within the first two years of life. rhIGF-I (mecasermin) has been reported to improve metabolic control and increase lifespan in DS patients. A case report and literature review were completed. We present a case involving a male patient with DS, harbouring a homozygous mutation in the INSR gene (c.591delC). Initial rhIGF-I application via BID (twice daily) injection was unsatisfactory, but continuous subcutaneous rhIGF-I infusion via an insulin pump improved weight development and diabetes control (HbA1c decreased from 10 to 7.6%). However, our patient died at 22 months of age during the course of a respiratory infection in in Libya. Currently available data in the literature comprising more than 30 treated patients worldwide seem to support a trial of rhIGF-I in SIR. rhIGF-I represents a treatment option for challenging SIR cases, but careful consideration of the therapeutic benefits and the burden of the disease is warranted. Continuous application via pump might be advantageous compared to single injections.

## 1. Introduction

The etiology and clinical presentation of severe insulin resistance is highly variable [1], with severe insulin receptor (INSR)-related insulin resistance syndromes (SIR) exhibiting a peculiar phenotypical spectrum. Within SIR, there is a continuum of insulin resistance, ranging from Donohue syndrome (DS) with no remaining insulin receptor function to the milder phenotypes of Rabson–Mendenhall syndrome (RMS) and type A insulin resistance [2,3].

Donohue syndrome was initially described in 1948 and 1954 by Donohue and Uchida [4]. Patients with DS exhibit severe intrauterine and postnatal growth retardation. Infants present with typical facial feature, resembling Leprechaun’s elves of Irish fairy tales, with lipoatrophy, acanthosis nigricans, hypertrichosis and severe hyperinsulinism, postprandial hyperglycemia and fasting hypoglycemia (for further clinical characteristics see Table 1). Most children with DS die within the first two years of life, mostly during the course of intercurrent infections of the upper airways, hypoglycemia or cardiomyopathy [3,5,6,7,8]. To date, there is no causative therapy for this very rare disease available (prevalence of DS < 1:1 Mio [9]). Rabson–Mendenhall syndrome was first described 1956 in three siblings with dental and skin abnormalities [10]. Children suffering from Rabson–Mendenhall syndrome typically show a milder phenotype [1], including impaired growth, abnormal nails and dentition as well as insulin resistance with acanthosis nigricans and hirsutism. At birth, patients with RMS also show fasting hypoglycemia due to severely increased insulin levels, but with progression of the disease, insulin levels decline [11]. In contrast to DS, patients develop recurrent diabetic ketoacidosis and microvascular complications during the second decade of life [2,5].

Diabetic ketoacidosis does not seem to occur in patients with Donohue syndrome [12], at least not in the first years of life [13]. Type A insulin resistance represents the least severe end of the spectrum [2]. It is usually diagnosed in teenagers or young adults who are not obese, but have severe insulin resistance, acanthosis nigricans, and hyperandrogenism in females. In some, but not all cases, a heterozygous genetic defect in the INSR gene has been reported [3].

Any condition of severe insulin resistance is therapeutically challenging with only limited treatment options being available. High dose insulin [14,15] and metformin [16] have been used in patients with insulin resistance syndromes. Metreleptin has been reported to improve blood glucose levels individuals with Rabson–Mendenhall syndrome [17,18].

Due to the structural similarity of insulin, proinsulin, insulin-like growth factor-I (IGF-I) and IGF-II and resemblances of the insulin and IGF-I receptor, IGF-I was proposed as another treatment option for patients with severe insulin resistance [19,20,21]. IGF-I use was first reported in the 1990s as an experimental treatment for patients with SIR, mostly with type A insulin resistance [22,23,24,25,26,27,28]. In the majority of the available case descriptions of SIR, an improvement in glucose homeostasis and a reduction in HbA1c was reported [21]. In addition to type A insulin resistance, mecasermin treatment of few patients with congenital insulin resistance syndromes with biallelic mutations within the insulin receptor gene (INSR) has been reported to improve glycemic control and weight gain [29,30,31,32] with variable results. In addition to the treatment option for SIR, rhIGF-I has been used previously in patients with growth hormone insensitivity syndrome [33,34,35,36,37,38,39,40,41,42]. Since 2008, rhIGF-I has been approved for the treatment of so-called “severe primary IGF-I deficiency” (SPGFD) [43,44].

This paper adds a case description, in which mecasermin treatment via an insulin pump successfully improved glucose homeostasis in a patient with DS and gives an update on the currently available knowledge on the clinical picture, treatment options and complications of these rare insulin resistance syndromes.

## 2. Materials and Methods

A case report and review of existing knowledge regarding the efficiency and safety of mecasermin in insulin receptor-related severe insulin resistance syndromes were completed using a systematic literature review with utilization of PRISMA (Preferred Reporting Items for Systematic Reviews and Meta-analyses) guidelines. We focused on the treatment of patients with DS, and performed a literature research in the Pubmed database using the following search terms:

“mecasermin”, “insulin resistance and recombinant IGF-I”, “Insulin resistance and recombinant IGF1”, “Donohue syndrome and IGF-I”, “Donohue syndrome and IGF1”, “Rabson–Mendenhall syndrome and IGF-I”, “Rabson–Mendenhall syndrome and IGF1”, “type A insulin resistance and IGF-I”, type A insulin resistance and IGF1”.

Thereby, we identified 189 records through database searching and 49 additional records which were identified through other sources, e.g., reference lists of other articles. The removal of duplicates led to 112 remaining records. After screening these records, 81 full-text articles were assessed for eligibility.

All articles were included in the synthesis of this paper. Because all studies reported only small numbers or presented single cases, a systematic meta-analysis could not be performed.

## 3. Case Report

We report on a male infant, who was born at term with a birth weight of 1300 g (−3.86 standard deviation scores; SDS) in Tripolis, Libya. At birth, typical dysmorphic features of DS could already be observed. In particular, muscle hypotonia and hyperglycemia with associated hyperinsulinism led to a clinical suspicion of an insulin receptor (IR)-related severe insulin resistance syndrome. His parents stated that they were very distantly related but were unable to provide more detailed information. Molecular analysis revealed a homozygous mutation of the insulin receptor gene in exon 2 (c.591delC). This frameshift mutation led to a nonsense mRNA and to a non-functioning insulin receptor, explaining the clinical picture of DS. Initial therapy consisted of a trial with metformin and insulin (no dosages available), which did not exert a relevant effect on glucose homeostasis. The patient presented first at our hospital at an age of 8 months, with a weight of 4020 g (<1st percentile, −5.5 SDS) and a height of 56 cm (<1st percentile, −5.61 SDS). He was in poor general health and in an extremely dystrophic nutritional state. Despite reduced subcutaneous fat tissue; he exhibited pronounced acanthosis nigricans and hypertrichosis. His skin texture throughout his whole body was remarkably dry and thick. Biochemical analysis revealed an HbA1c concentration of 10.0% (86 mmol/mol), with very high insulin (>1700 mU/L) and C-peptide (>4.5 ng/mL) levels, accompanied by undetectable IGF-I (<−25 ng/mL) and IGFBP-3 (<0.5 μg/mL) concentrations.

An abdominal ultrasound demonstrated hyperechogenic kidneys. Echocardiography showed a small, but hypertrophic left ventricle. Ophthalmological examination and otolaryngeal status, including auditory brainstem response audiometry, was without pathological findings, except for mucous obstruction of the upper airways.

Based on previous publications of patients suffering from either type A insulin resistance or DS, who were successfully treated with rhIGF-I [1,12,30], we decided to start a mecasermin trial. The parents’ informed consent was obtained after they were given detailed information on the therapy and medication.

**Figure 1:**
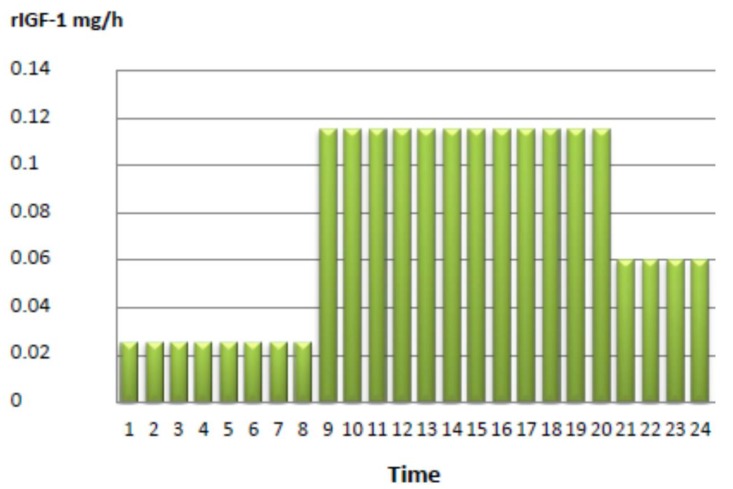
Mecasermin delivery in mg/h via an insulin pump in the reported patient.

The initial treatment consisted of rhIGF-I application via BID injection. The initial dosage consisted of 2 × 200 μg mecasermin, which was escalated to a final dose of 2 × 1 mg/day, corresponding to a dosage of 0.5 mg/kg body weight per day over a period of one week. However, since we found no relevant effect on diurnal glucose control, and rather, an aggravation of fasting hypoglycemia during the night, we decided to test continuous subcutaneous mecasermin infusion using an insulin pump. Mecasermin was diluted with physiological NaCl infusion solution, with a final rhIGF-I dosage of 1.8 mg/day (see Figure 1).

After discharge, the family travelled back to Libya. Because of the distance to the family’s home country and the difficult political situation in Libya, it was not possible to see the patient and his parents more frequently than every four to six months. During these follow-up examinations anthropometry, clinical status, abdominal and thymus ultrasound, bone age, echocardiography, otolaryngeal and ophthalmological status as well as several biochemical analyses (IGF-I, IGFBP-3, glucose homeostasis, insulin level, HbA1c, cholesterol, growth hormone, TSH, ft4, prolactin, blood count, electrolytes, renal and liver parameters) were monitored. In between visits, we had regular email correspondence with the parents, and the child was seen by a diabetologist in his home country.

**Figure 2:**
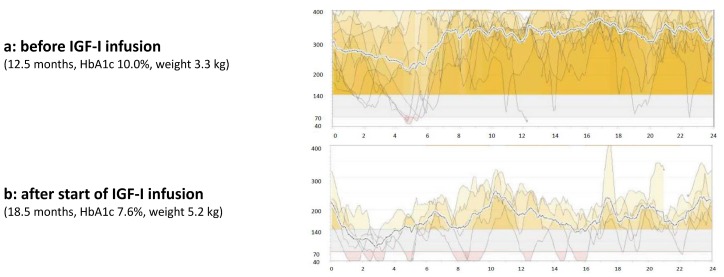
Glucose profile using a continuous glucose monitoring system (CGMS) before (**a**) and after (**b**) initiation of continuous subcutaneous mecasermin infusion via an insulin pump.

We saw the patient again at an age of 13 months. Even though the family was highly motivated to use the rhIGF-I infusion, intermittent therapy was not possible because the family could not get the medication in their home country due to logistical problems. Meanwhile the patient lost weight, now weighing 3300 g. His HbA1c was still 10%. We reinitiated the therapy, and this time the family took the medication from Germany to Libya for the next 6 months. At an age of 18 months, after completion of six months of continuous subcutaneous rhIGF-I infusion via insulin pump, the patient presented again in our hospital. His weight was 5250 g (−5.65 SDS). His HbA1c had improved from 10.0 to 7.6%. Blood glucose trends were more stable than they had been without mecasermin therapy (see Figure 2).

In addition, we saw a moderate improvement in motor function and muscular strength. The renal ultrasound, echocardiography and ophthalmological examination results were unchanged relative to those collected before treatment with mecasermin. The otolaryngeal examination revealed adenoid hypertrophy as a potential adverse event of rhIGF-I therapy, leading to an intermittent nocturnal oxygen requirement. Adenoidectomy and tympanic paracentesis were performed. Postoperatively, weaning from ventilation and extubation was complicated by rapid desaturation and hypoxia, requiring cardiopulmonary reanimation (CPR) and subsequent ventilation for several days. Following stabilization, the patient was discharged, and continued with continuous rhIGF-I infusion as described above. Unfortunately, four months after discharge at an age of 22 months the patient died during the course of a respiratory infection in a hospital in Libya.

## 4. Overview of Current Knowledge on the Use of rhIGF-I in SIR

### 4.1. Clinical Picture of SIR

As described above, INSR-related severe insulin resistance compromises a phenotypical continuum, with the most severe phenotype seen in DS (leprechaunism) and a milder phenotype in RMS [3], with type A insulin resistance being a relatively mild form of SIR. The INSR gene maps to the short arm of chromosome 19 and is composed of 22 exons [45].

DS and RMS are caused by autosomal recessive mutations of the INSR gene. There is a limited correlation between genotype and phenotype [45]. The most severe phenotypes result from mutations that markedly impair insulin binding. Mutations in the insulin receptor that retain residual insulin-binding are correlated with prolonged survival [45,46]. However, a definitive genotype–phenotype correlation for INSR defects is difficult to establish, primarily due to the rarity of these syndromes [46]. Dysmorphic features of DS and RMS resemble each other (see Table 1), but are less apparent in RMS. Some clinical aspects, like hirsutism or genital enlargement sometimes seem to appear later in RMS [3].

Children with DS present frequently with severe global developmental delay [8], which might be caused by recurrent severe hypoglycemic episodes [47]. Without intervention, death occurs within the first two years of life. Respiratory infections, hypoglycemia [5,6] and cardiomyopathy [7,8] seem to be the major causes of death [3]. Patients who were characterized as leprechaunism and showed long-term survival and normal psychomotor development [48] might have had benefit from early rhIGF-I therapy or should rather have been classified as a patient with RMS [3].

The manifestation of type A insulin resistance occurs commonly around puberty. Patients are normally not obese (in contrast to the majority of polycystic ovary syndrome (PCOS) subjects) and suffer from hyperandrogenism (PCOS in females, hirsutism, acne) and insulin resistance (diabetes, acanthosis nigricans). In subjects with type A insulin resistance, growth in infancy and childhood is normal. There is no intellectual impairment [12,49] (Table 1). Some cases can be attributed to a detectable heterozygous genetic defect in the INSR gene (most cases are autosomal-dominant, but autosomal-recessive cases have been reported as well).

Simpkin et al. [50] reported about 17 patients (eight with a complete dataset) with INSR mutations and a clinical diagnosis of DS or RMS. INSR dysfunction was associated with hypercalciuria and nephrocalcinosis, but no other consistent abnormality of renal function was noted. These results were in contrast to results in genetically modified mice, which showed elevated blood pressure and progressive diabetic nephropathy [51]. Differences between mice and men have also been observed for other aspects of the phenotype of insulin receptor deficiency [50]. INSR knockout mice die soon after birth because of ketoacidosis [52], whereas diabetic ketoacidosis does not seem to occur in human patients with Donohue syndrome [53], at least not in the first years of life [13]. Complete deletion of the insulin receptor gene is compatible with life in men [54].

To achieve the best possible treatment of children with DS and RMS, a close collaboration between several subspecialties is required. Paediatric endocrinologists are typically in charge of assessing glycemic control and respective treatment regimens, paediatric cardiologists monitor hypertrophic cardiomyopathy, and neuropaediatricians are involved in monitoring neurological development and the initiation of supportive care. A screening protocol regarding the occurrence of nephrocalcinosis and/or impaired renal function as well as liver function should be initiated. Adequate nutrition and caloric intake are of paramount importance to improve blood sugar concentrations, weight gain and growth. Routine ultrasound examinations should be performed to control the morphology of ovaries in girls as well as the morphology of the kidneys, liver and spleen in both sexes [3]. In subjects in whom rhIGF-I treatment is started, repetitive fundoscopy and otolaryngeal examinations should be scheduled. Because of dental crowding, orthodontic treatment could be necessary [55,56].

### 4.2. Treatment Options in SIR Other Than rhIGF-I

Hyperglycaemia in patients with insulin receptor mutations is extremely difficult to treat [12,18], and patients are at risk for early morbidity and mortality from the microvascular complications of diabetes [18,57]. In particular, DS has a poor prognosis with only few therapeutic options being available. In subjects with type A insulin resistance or RMS, the use of insulin sensitizing drugs, such as metformin or rosiglitazone, should be first-line therapy. However, previous reports have indicated that a positive therapeutic impact is only be observed in a fraction of patients and that, over time, the effect of these drugs seems to diminish [64]. Frequently, affected patients require multidrug therapy [64,65].

Moreira et al. [65] reported a successful reduction of HbA1c in a patient with RMS using a combination of nutritional counseling, physical activity, and the following medications: metformin, pioglitazone, vildagliptin and acarbose. The authors mentioned that the use of dipeptidylpeptidase-4 inhibitors might be a promising new approach for patients with RMS.

In cases for whom insulin therapy is initiated, patients require very high insulin doses and therefore, in most cases, U-500 insulin is used for treatment [12,14,66,67]. In patients treated with U-500 insulin, continuous subcutaneous insulin infusion (CSII) is a viable option [12]. While common therapeutic targets may not be achievable in SIR patients, large doses of insulin can improve hyperglycemia, catabolic state and weight loss and reduce the risk of microvascular complications [14]. Metreleptin has been reported to improve glucose metabolism in subjects with genetic lipodystrophic syndromes, and it has been reported to be a potential therapeutic option in patients with RMS [17,18]. In that regard, Brown et al. [18] treated five patients with RMS with metreleptin and were able to show a decrease in HbA1c, from 11.4% at baseline to 9.3% after six months and 9.7% after 12 months of metreleptin treatment. However, the authors also reported that these beneficial effects of metreleptin on HbA1c concentration appeared to wane over time, and metreleptin treatment, with or without insulin, in the long-term was insufficient to achieve targeted HbA1c levels. There has been a single observation in a patient with type A insulin resistance available which reported a remarkable improvement of glycemic control after starting a suppressive dose of levothyroxine [68]. Since the thyroid hormone is an activator of brown adipose tissue and because positron emission tomography (PET) showed the presence of brown adipose tissue in this patient, the activation of brown adipose tissue might be a new therapeutic target of SIR [69]. In DS, metformin and insulin treatment usually do not improve glycaemic control [12,57]. To the best of our knowledge, successful metreleptin treatment has not been reported in DS yet. Frequent or continuous gastric feeding seems to be efficient for preventing fasting hypoglycaemia in patients with DS [12]. Table 2 summarizes the different treatment options in patients with SIR.

### 4.3. Use of rhIGF-I in Insulin Receptor-Related Severe Insulin Resistance Syndromes

**Figure 3:**
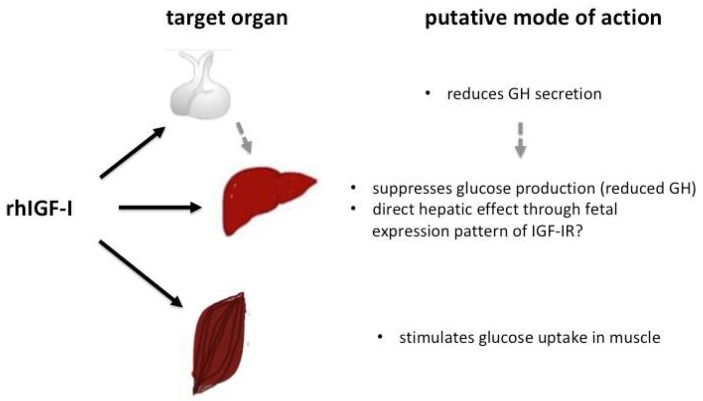
Putative effects of rhIGF-I on glucose homeostasis.

Because of its previously reported effect on glucose homeostasis, rhIGF-I has been postulated as a potential therapeutic option in SIR syndromes. The IGF-I gene is a major target of growth hormone (GH) and an important mediator of GH-stimulated growth, but exerts different effects on carbohydrate, lipid and protein metabolism compared to GH. IGF-I stimulates a reduction in blood glucose and circulating insulin levels and therefore, can cause hypoglycaemia [70]. This effect is in part mediated through IGF-I’s structural similarity with insulin and its affinity to the insulin and IGF-I receptors, which share common post-receptor signaling pathways [19,20]. In vitro studies using adenoviral IGF-I gene transfer into ß-cells have shown the promotion of ß-cell survival and proliferation [71]. Furthermore, islet cell exposure towards IGF-I seems to sustain or increase insulin synthesis [72]. Therefore, an increase in the circulating insulin concentration under conditions where a residual insulin effect is maintained, either through insulin/IGF-hybrid receptors or through mutated insulin receptors with residual IR function, might lead to an improvement in glucose homeostasis. In SIR, the effects of rhIGF-I on glucose homeostasis are thought to occur mainly through increased glucose uptake into peripheral tissues (e.g., muscle) and a reduction in hepatic glucose production (see Figure 3).

The first reports of rhIGF-I use in patients with SIR were published in the 1990s. Single-dose intravenous administration of rhIGF-I with a dosage of 100 μg/kg/day was associated with declines in blood glucose, plasma insulin, C-peptide and GH concentrations [23,24]. These initial trials were followed by trials using subcutaneous application of rhIGF-I [22,25,26,27,28,63,73]. Kuzuya [73] first described the usage of rhIGF-I over a period of several months (up to 16 months) as leading to a decrease in HbA1c concentration in patients with SIR. Most publications are either single case reports or include only few (up to six) patients with an SIR syndrome, with pronounced differences in clinical phenotypes (from DS over RMS to Type A IR). Therefore, a direct comparison of the respective treatment effects between the mentioned case reports/small studies is not possible. Table 3 gives a summary of different rhIGF-I trials reporting either short or long-term effects of rhIGF-I in SIR, including trials with no apparent benefit of rhIGF-I in the treatment of patients with SIR syndromes [74,75]. Backeljauw [75] and coworkers treated two infants classified as DS with intravenous rhIGF-I over a period of 66 and 62 h, respectively, and could not see any apparent glucose lowering or a decrease in insulin concentration. A potential explanation for this might be that the effect of rhIGF-I on glucose metabolism in SIR is just as heterogenous as the syndrome itself [74]. In addition to SIR, there are some single case reports regarding successful long-term use of rhIGF-I in patients with leprechaunism [29,31]. In the patient reported above, we were not able to see an improvement of glucose homeostasis by administration of BID mecasermin application. However, we cannot exclude that a higher dosage or more prolonged trial of mecasermin application might have led to a more favourable response [29,31,32] (see also Table 3). Still, the observation of more satisfactory results on blood glucose homeostasis using a continuous application of rhIGF-I via an insulin pump is in accordance with other reports [30,76]. The half-life of insulin-like growth factor I seems to be decreased in patients with insulin receptor defects, which, in part, can be explained by drastically decreased or absent IGF binding protein-3 concentrations. In order to analyse why rhIGF-I is not consistently effective in patients with Rabson–Mendenhall syndrome, Longo et al. [63] measured IGF-I concentrations in a patient after subcutaneous injections. The IGF-I half-life was dramatically reduced (1.3–3 h) compared to the normal range of 17–22 h in healthy subjects. This might partly explain why the use of “moderate” mecasermin dosages of 0.5 mg/kg/day, or even less via continuous subcutaneous infusion, can lead to results that are as satisfactory as those from the use of a very high dose of rhIGF-I through a single injection (for example 1.6 mg/kg/day [29]).

In addition to pharmacokinetic influences on treatment success, functional analyses of the INSR gene can aid in the prediction of the severity of an INSR mutation and its associated disease phenotype [76] and thereby, might aid in predicting the effectiveness of rhIGF-I treatment. Furthermore, the timing of treatment has been associated with the treatment response. Early treatment with rhIGF-I has been reported to increase the lifespan of patients with DS or Rabson–Mendenhall syndrome [29,76]. However, the effectiveness of rhIGF-I therapy seems to decrease with progression of the disease [77].

Mecasermin use has been associated with a number of adverse effects, in particular hypoglycaemia, pain at injection side and lymphoid tissue hypertrophy. Lymphoid tissue hypertrophy has been described in about 25% of all patients who were treated with mecasermin and is associated with the requirement of surgery in >10% of patients [20,80] (see Table 4).

In our case report, after six months of mecasermin treatment, our patient underwent surgery for probably treatment-related development of adenoid hypertrophy. Nakae, Kitamei and Jo et al. [29,77,78] reported information about one patient at different time points over the clinical course. The patient was treated with rhIGF-I from an age of six months until 18 years, with further follow-up to an age of 24 years. During this time course, the patient developed several symptoms, which could be interpreted as adverse effects of rhIGF-I therapy, although disease-inherent development of the complications cannot be ruled out: tonsillar hypertrophy (at three years of age), retinal neovascularization (at 12 years of age), mastitis (at 16 years) and endometrial cancer (at 24 years of age). In another patient with DS [30], the development of a granulosa cell tumor was reported, but its relationship to concomitant rhIGF-I therapy remains unclear, since a comparable tumour developed in a girl with leprechaunism who was not treated with rhIGF-I [81]. Other authors did not observe any side effects of rhIGF-I therapy in patients with SIR over a period of five [31] or 10 years [48]. Table 4 summarizes the possible adverse effects of mecasermin therapy in general and the reported side effects of its use in SIR.

In addition to the putative adverse effects depicted in Table 4, children with DS seem to have an increased risk during general anaesthesia [49]. The increase in risk might be related to the presence of restrictive lung disease, abdominal distension and difficult airway conditions. Kirkwood presented a case series of five patients with DS who underwent 12 treatments with general anaesthesia. Two anaesthesia treatments (in two patients) were complicated by cardiac arrest, secondary to difficult manual ventilation, intubation, and hypoxia and required CPR, resembling our case report.

## 5. Conclusions

The treatment of SIR syndromes is challenging. Even though there are some treatment options available for patients with a Type A insulin resistance phenotype—the mildest form of the INSR-related SIR syndromes—general treatment goals, e.g., long-term normalization of HbA1c cannot be achieved in most cases. The effects of all medications mentioned above (insulin sensitizer, insulin, metreleptin, mecasermin) seem to diminish over time.

The optimal timing, application form and dosage of rhIGF-I still remain unclear, but this seems to be the most effective type of therapy in patients with DS so far. Continuous application via pump might be advantageous compared to single injections.

Further treatment options are clearly needed to improve the quality of life and life expectancy of patients with SIR syndromes. Careful consideration of the therapeutic benefits and the burden of the disease is warranted.

## Figures and Tables

**Table 1:** Characteristic clinical features of subjects with Donohue syndrome (DS), Rabson–Mendenhall Syndrome (RMS) and Type A Insulin Resistance (Type A IR) [3,5,6,7,8,10,49,50,56,57,58,59,60,61,62,63].

	DS	RMS	Type A Insulin Resistance
**Molecular genetics**	Homozygous mutation in the insulin receptor (INSR) gene	Compound heterozygous mutation in INSR gene	Mutation in the insulin receptor gene (autosomal-dominant or autosomal-recessive)
**Face**	Proptosis	Resembling DS	Normal phenotype
	Infraorbital folds	or milder phenotype	
	Large, posteriorly rotated ears		
	Thick lips		
	Gingival hyperplasia		
	Broad nasal tip		
**Other**	Large hands and feet (relative to body) Gingival hypertrophy	Early dentition and dental crowding Nail dysplasia	Usually not obese
	Abdominal distension		
	Reduced lateral thoracic dimension		
	Hyperplasia of nipples	Hyperplasia of nipples	
	Genital enlargement	Genital enlargement	
	Intrauterine growth restriction	Growth retardation (less severe than in DS)	Normal growth
	Postnatal failure to thrive		
**Organ pathologies**	Organomegaly (kidney, liver, spleen)	Organomegaly (kidney, liver, spleen)	
	Hypertrophic cardiomyopathy (HCOM)	Hypertrophic cardiomyopathy	
	Nephrocalcinosis Renal tubular dysfunction	Nephrocalcinosis Renal tubular dysfunction	
	Enlarged polycystic ovaries	Enlarged polycystic ovaries	Polycystic ovaries
	Rectal prolapse	**Second decade:** microvascular complications	
	Cholestasis	Retinopathy Peripheral neuropathy Renal vascular complications	
**Skin Features**	Hypertrichosis	Hypertrichosis	Hirsutism
	Acanthosis nigricans	Acanthosis nigricans	Acanthosis nigricans
	Hyperkeratosis		
	Thick, hyperelastic skin		
	Dry skin		
	Decreased subcutaneous fat mass		
**Biochemical Findings**	Hyperinsulinemia	Same as DS in first year	Hyperinsulinemia or
	Extremely elevated plasma insulin and C-peptide levels	of live Insulin level decline steadily	extreme resistance to exogenous insulin
	Hyperglycemia	Resulting in increased	Hyperandrogenism
	Fasting hypoglycemia	glucose levels, fewer hypoglycemic events	Increased serum testosterone
	Absence of ketoacidosis	Risk of ketoacidosis	
	Decreased IGF-I and IGFBP-3 serum concentrations Hypercalciuria	Decreased IGF-I levels and IGFBP-3 Low triglyceride levels, high HDL levels Hypercalciuria	
**Neurological Findings**	Severe global developmental delay	Variable developmental	No general impairment
	Axial hypotonia	delay to normal	
	Muscle atrophy	Intelligence	
**Life expectancy**	Usually death within first two years of life, due to intercurrent infections, severe hypoglycemia, cardiomyopathy	Usually death within second decade of life, due to ketoacidosis or microvascular complications	

**Table 2:** Therapeutic efficiency of available treatment options in SIR.

Treatment Option	DS	RMS	Type A IR
Nutrition:			
Frequent/continuous feeding	Prevents fasting hypoglycaemia	Prevents fasting hypoglycaemia	
Avoidance of high carbohydrate diet		Useful	Useful
Insulin sensitizer	No effect	Early usage recommended. Improvement of hyperglycaemia	Early usage recommended. Improvement of hyperglycaemia
High dose insulin	No effect	Improvement of hyperglycaemia, catabolic state, weight loss and microvascular complications [14]	Improvement of hyperglycaemia, catabolic state and microvascular complications [14]
Metreleptin	No assessment available	Improvement of HbA1c [18]	No study with type A IR patients available.
Mecasermin (rhIGF-I)	See Table 3	See Table 3	See Table 3

**Table 3:** Treatment trials with rhIGF-I in patients with severe INSR-related insulin resistance syndromes (SIR).

Author	Treatment Duration of rhIGF-I	SIR Syndrome (n)	Dosage	Efficacy and Safety
Quin et al., 1990 [23]	Short term 1 dose	RMS (1 patient)	100 μg/kg/day i.v.	Blood glucose, plasma insulin, C-Peptide and growth hormone (GH) declined
Schoenle et al., 1991 [24]	Short term 2 doses	Type A IR (3 patients)	100 μg/kg/day i.v.	Blood glucose, plasma insulin and C-peptide decreased
Hussain et al., 1993 [22]	Short term 4 days, BID	Type A IR (1 patient)	160 μg/kg/day s.c.	Lowering of fasting and postprandial blood glucose, insulin and C-peptide levels
Kuzuya et al., 1993 [73]	Up to 16 months	Type A IR (6 patients) DS (2 patients) Congenital lipodystrophy (2 patients) Other (1 patient)	100 to 400 μg/kg/day	Lowering of fasting and postprandial glucose. Decrease of HbA1c and fructosamine
Morrow et al., 1994 [27]	3–4 weeks	Type A without insulin receptor mutations (2 patients)	100 μg s.c.	Reduction of blood glucose level, enhancement of insulin sensitivity
Backeljauw, 1994 [75]	66 h and 62 h	DS (2 patients)	Up to 110 μg/kg/h and 40 μg/kg/h i.v.	No apparent glucose lowering effect, decrease in insulin concentration
Zenobi, 1994 [25]	5 days	Type A IR (2 patients)	150 μg/kg 2x/day s.c.	Decrease of fasting blood glucose. Decrease in fasting insulin and C-peptide by 65%
Longo et al., 1994 [63]	16 months	RMS (1 patient)	Up to 100 μg/kg/day s.c.	No significant effect on glycaemic control and growth
Nakashima et al., 1995 [28]	9 months	Type A IR (1 patient)	0.08–0.2 mg/kg/day s.c.	Decrease in blood glucose level, free fatty acid concentration, HbA1c; enhanced insulin sensitivity, improvement of acanthosis nigricans
Vestergaard et al., 1997 [26]	2 weeks high dose rhIGF-I	SIR (4 patients)	60 μg/kg 2x/day s.c.	Reduction of fasting blood glucose and insulin
	10 weeks low dose rhIGF-I	SIR (3 patients)	40 μg/kg/day s.c.	
Takahashi et al., 1997 [32]	6 months	Leprechaunism—1 patient	100 μg/kg/day up to 1000 μg/kg/day	Fasting blood glucose, insulin, HbA1c, body weight and development improved
Nakae et al., 1998 [29]	6 years and 10 months	DS (?) or RMS (?) (1 patient at different time points)	Up to 1.6 mg/kg/day	Maintained growth rate, HbA1c near normal range
Kitamei et al., 2004 [78]			Intermittent and continuous s.c injection	Adult height was 143 cm (−2.7 SDS for Japanese girls)
Jo et al., 2013 [77]	Withdrawl of rhIGF-I treatment at 18 years, due to diabetic ketoacidosis and start of high dose insulin			Recurrent episodes of ketoacidosis. HbA1c up to 12–13%
Regan et al., 2010 [79]	16 weeks	Type A IR (5 patients)	0.5–2 mg/kg rhIGF-I/rhIGFBP-3	HbA1c improvement, reduction of acanthosis nigricans
Weber et al., 2014 [30]	16 months (from 19 months up to 35 months—death of the patient)	DS (1 patient)	BID 80 up to 640 μg/kg/day s.c.; At 30 months: continuous s.c. infusion via insulin pump: 800–1200 μg/kg/day	HbA1c improvement from 9.5 to 7.7%, rebound to 9.8% Improvement of HbA1c from 9.8 to 8.8%, Moderate weight gain
De Kerdanet et al., 2015 [48]	8.7 years; 2 years	DS (1 patient)	IGF-I/IGFBP3 combination, subsequ. IGF-I alone 50 μg/kg/day s.c.	Decrease in mean glycaemia with large variation. Improvement of growth.
Carmody et al., 2016 [31]	5 years	RMS (1 patient)	rhIGF-I up to 1.72 U/kg 2x/day s.c. + metformin	Decrease in insulin, homa index and HbA1c. Growth maintained within target height range.

**Table 4:** Adverse effects of mecasermin treatment in conditions other than SIR and in patients with SIR.

Adverse Effects	Indication Other than SIR [20]	SIR
Hypoglycaemia	50–86%	+++
Mild pain/erythema at injection side	+++	++ [27,79]
Paresthesia and painful toes		++ [28]
Lipohypertrophy	1/3	
(Asymptomatic) tachycardia	+++	+
Parotid swelling	+	
Facial nerve palsy	+	
Retinopathy/worsening of retinopathy	+	+ [1,20,28,77,78,79] [77,78] ^#^ (patient’s age 12 years)
Muscle pain *	+	+ [1,20]
Fluid retention/edema *	+	+ [1,20]
Arthritis *	+	+ [20]
Benign intracranial hypertension /papilledema	5%	+ [1]
Lymphoid tissue hypertrophy: tonsillar hypertrophy Adenoidal hypertrophy	25% (>10% require surgical intervention)	+ [29] ^#^ (patient’s age 3 years) + [76]
Thymic hypertrophy	35% (X-ray)	
Mastitis		? [77] ^#^ (age 16 years)
Endometrial cancer		+ [77] ^#^ (age 24 years)

* dose related, high doses were given. ^#^ Nakae, Kitamei and Jo et al. report information about the same patient at different ages. +: rarely reported, ++: frequently reported, +++: common feature.
